# Synthesis and Characterization of Titanium Dioxide Hollow Nanofiber for Photocatalytic Degradation of Methylene Blue Dye

**DOI:** 10.3390/membranes11080581

**Published:** 2021-07-30

**Authors:** Nurul Natasha Mohammad Jafri, Juhana Jaafar, Nur Hashimah Alias, Sadaki Samitsu, Farhana Aziz, Wan Norharyati Wan Salleh, Mohd Zamri Mohd Yusop, Mohd Hafiz Dzarfan Othman, Mukhlis A Rahman, Ahmad Fauzi Ismail, Takeshi Matsuura, Arun M. Isloor

**Affiliations:** 1 Advanced Membrane Technology Research Centre (AMTEC), School of Chemical and Energy Engineering, Faculty of Engineering, Universiti Teknologi Malaysia, 81310 UTM Johor Bahru, Johor, Malaysia; nnatasha6@live.utm.my (N.N.M.J.); farhana@petroleum.utm.my (F.A.); hayati@petroleum.utm.my (W.N.W.S.); zamriyusop@utm.my (M.Z.M.Y.); hafiz@petroleum.utm.my (M.H.D.O.); mukhlis@petroleum.utm.my (M.A.R.); afauzi@utm.my (A.F.I.); 2 Department of Oil and Gas Engineering, School of Chemical Engineering, College of Engineering, Universiti Teknologi MARA, 40450 Shah Alam, Selangor, Malaysia; nurhashimah@uitm.edu.my; 3 National Institute for Materials Science, 1-2-1, Sengen, Tsukuba 305-0047, Japan; samitsu.sadaki@nims.go.jp; 4 Industrial Membrane Research Laboratory, Department of Chemical Engineering, University of Ottawa, Ottawa, ON K1N 6N5, Canada; matsuura@uottawa.ca; 5 Membrane and Separation Technology Laboratory, Department of Chemistry, National Institute of Technology Karnataka, Surathkal, Mangalore 575 025, India; isloor@yahoo.com

**Keywords:** titanium dioxide, hollow nanofibers, electrospinning, template synthesis, photocatalysis and photocatalytic, bisphenol A

## Abstract

Environmental crisis and water contamination have led to worldwide exploration for advanced technologies for wastewater treatment, and one of them is photocatalytic degradation. A one-dimensional hollow nanofiber with enhanced photocatalytic properties is considered a promising material to be applied in the field. Therefore, we synthesized titanium dioxide hollow nanofibers (THNF) with extended surface area, light-harvesting properties and an anatase–rutile heterojunction via a template synthesis method and followed by a calcination process. The effect of calcination temperature on the formation and properties of THNF were determined and the possible mechanism of THNF formation was proposed. THNF nanofibers produced at 600 °C consisted of a mixture of 24.2% anatase and 75.8% rutile, with a specific surface area of 81.2776 m^2^/g. The hollow nanofibers also outperformed the other catalysts in terms of photocatalytic degradation of MB dye, at 85.5%. The optimum catalyst loading, dye concentration, pH, and H_2_O_2_ concentration were determined at 0.75 g/L, 10 ppm, pH 11, and 10 mM, respectively. The highest degradation of methylene blue dye achieved was 95.2% after 4 h of UV irradiation.

## 1. Introduction

Water contamination caused by domestic wastes, industrial chemicals, fertilizers, and organic dyes has become one of the global environmental concerns [1]. Organic dyes are being used in industries like leather, cosmetics, textile, and paper manufacturing. The amount of dye produced annually in the world was estimated to be a million tons [2] and the wastewater from these industries is often discharged into natural waters without being treated. The pigmented, highly hazardous, non-biodegradable, and carcinogenic dye-contaminated effluents may affect the appearance of water and cause serious diseases in humans even at a very low concentration. Thus, it is essential to develop an effective and reliable method to treat dye wastewater. Typically, conventional treatments like adsorption [3] and coagulation [4] are used in treating dye-contaminated wastewater. However, these methods do not completely destroy the pollutants but produce suspended particles or sludge that require post-treatment disposal.

In recent years, photocatalytic oxidation has been deemed a promising water purification technique. Essentially, photocatalytic oxidation involves the reaction between light-absorbing catalysts and pollutants. Photocatalytic degradation can occur within a few hours at room temperature. At the end of the process, the pollutants are mineralized to less harmful products (carbon dioxide and water) without forming secondary hazardous products. Metal oxide semiconductors like titanium dioxide, zinc oxide, silicon dioxide, and cerium dioxide [5] are often used as photocatalysts. Titanium dioxide (TiO_2_) is one of the most commonly used metal oxides owing to its high stability, low cost, abundance, stability, efficient photoactivity, and safety to human beings and the environment [6]. The basic principle of photocatalytic degradation needs a semiconductor whose electronic structure consists of a valence band (VB) and conduction band (CB), which are separated by a bandgap of energy. Upon the irradiation of light energy equal to or higher than the bandgap energy level, the valence electron (e^−^) is promoted to the valence band, leaving a positive hole (h^+^) in the valence band. Both e^−^ and h^+^ are strong reducing and oxidizing agents, respectively. They react with the water molecules to produce superoxide (•O_2_^−^) and hydroxyl (•OH) radicals, that attack the organic compounds, eventually converting them into harmless products of carbon dioxide and water.

Degussa P25, a commercially available TiO_2_, is one of the most efficient photocatalysts in terms of cost and photocatalytic ability, with a number of works published elsewhere [7,8,9,10,11]. Despite that, these studies reported some issues with this particular photocatalyst, and one of them is the high bandgap energy (~3.2 eV) The high bandgap value limits the light utilization efficiency and reduces the number of electrons that could participate in the redox reaction, which consequently deteriorates the photocatalytic activity. The photoexcited electrons that exist in a meta-stable state also have the tendency to recombine with the positive holes. Another drawback of Degussa P25 TiO_2_ reported is the low specific surface area (50 m^2^/g). The efficiency of a photocatalyst is highly dependent on its active surface area. The larger the amounts of reagents adsorbed on the surface, the higher their possibility to take part in the reaction.

A variety of strategies have been employed to enhance the photocatalytic properties of a semiconductor, including morphology control [12], doping [13], conjugation [14], and incorporation of co-catalyst [15]. The structure of semiconductors imposes a significant impact on the photocatalytic properties of a semiconductor; hence, nanostructure engineering has been acknowledged as an effective strategy. This can be proven through the application of various nanostructures photocatalytic fields such as quantum dots, nanowires, nanorods, nanofibers, and hollow nanofibers [16]. In contrast with solid one-dimensional nanostructures, hollow nanofibers are interesting to be further studied because they display desirable properties as photocatalysts, owing to their higher surface-to-volume ratio. A previous study reported that the surface area of the hollow nanofibers is twice as large as conventional nanofibers [17]. Thus, this study aims to enhance the photocatalytic properties of the TiO_2_ photocatalyst by synthesizing titanium dioxide hollow nanofibers (THNF) with extended surface area, maximized light adsorption ability and improved bandgap through the construction of anatase–rutile heterojunctions.

In recent years, many techniques have been reported for the fabrication of hollow nanofibers such as one-pot electrospinning [16], coaxial electrospinning [18], and self-assembly [19]. However, these methods suffer from some limitations. For example, there are limited common solvents that can be used to prepare a blend polymer solution. Moreover, it is required to find the optimum electrospinning parameters for each polymer in the blend solution. Meanwhile, for coaxial electrospinning, the core and sheath solution should be immiscible for the successful synthesis of hollow nanofibers. In the self-assembly process, the process is complex and has low throughput. The most prominent limitation of the above-mentioned technique is the inability to control the properties of the resultant structure in terms of dimensions, morphology, and crystallinity.

On the other hand, template synthesis enables the fabrication of hollow nanofibers with controlled properties. Typically, a precursor of the target material is deposited onto a sacrificial template, followed by a template elimination process [20]. Electrospun polymers are often used as the template material due to the simple preparation method, morphology flexibility, and very low cost. The morphology of the resultant product is directly influenced by the property of the template. Therefore, in this study, we proposed the preparation of TiO_2_ hollow nanofibers with controllable properties via template synthesis for the photocatalytic degradation of methylene blue. Polyacrylonitrile (PAN) was used as the template and the TiO_2_ precursor was deposited via the dip-coating method. Then, the effect of calcination temperature during the template removal on the fiber properties was determined. We also proposed the possible formation mechanism of THNF. To the best of our knowledge, the mechanistic insights of TiO_2_ hollow nanofiber formation via template synthesis have not been reported elsewhere. In addition, the effect of several operating parameters that are the catalyst loading, dye concentration, pH, and hydrogen peroxide concentration on the photodegradation of methylene blue (MB) dye was also investigated.

## 2. Materials and Methods

### 2.1. Materials

Polyacrylonitrile, PAN at MW = 150,000 and titanium (IV) isopropoxide, TTIP, (97% in solution) were bought from Sigma Aldrich. N,N-dimethylformamide (DMF) was bought from RCI Labscan. Meanwhile, acetic acid (glacial 100% purity) and nitric acid (65% purity) were bought from Merck. All materials were used directly without any purification.

### 2.2. Preparation of Nanofibers Template

PAN dope solution was prepared by dissolving 8 wt. % of PAN with DMF. The mixture was stirred at 60 °C for 24 h until homogenous. The electrospinning machine was supplied by Progene Link Sdn Bhd, Selangor, Malaysia. The prepared solution was fixed in a plastic syringe with a blunt tip stainless steel needle. A voltage of 12 kV was applied for electrospinning. The electrospinning process was conducted at the flow rate of 1.0 mL/h and 15 cm horizontal distance from the needle tip to the collector. After the electrospinning process was completed, the as-spun nanofibers were dried at 80 °C for 2 h to remove the excess moisture and solvent, followed by calcination. The oxidation process was done as follows: (i) calcination at 150 °C for 1 h (2 °C/min); (ii) calcination at 230 °C for 3 h (1 °C/min); and (iii) cooling to room temperature.

### 2.3. Synthesis of THNF Photocatalysts

THNF was obtained through three steps. The first one is the electrospinning of PAN nanofibers. Secondly, TiO_2_ sol-gel was deposited on the obtained as-spun PAN nanofibers. Lastly, TiO_2_/PAN nanofibers composite (TPNF) was calcined for template removal. TiO_2_ sol-gel was prepared by mixing 10 mL of TTIP and 10 mL acetic acid, CH_3_COOH. Then, 100 mL of distilled water was added to accelerate the hydrolysis reaction. A total of 1 mL of nitric acid, HNO3 was added into the mixture to achieve a pH range of 4–6. The mixture was stirred for 24 h at 60 °C until a whitish-blue solution was formed. TPNF composite was formed by dip-coating as-spun PAN nanofibers in the sol-gel at immersion and withdrawal speed of 5 mm/s. Then, the nanofibers were dried in the oven at 90 °C for 2 h before being calcined in air at 600 °C for 4 h to remove the PAN component. During the calcination process, the calcination temperature was set at 400, 500, and 600 °C for 4 h. The samples were denoted as THNF400, THNF500, and THNF600, respectively.

### 2.4. Characterization

The morphology of the nanofibers was observed using a field emission scanning electron microscope (FESEM, ZEISS Crossbeam 340, Jena, Germany). Thermogravimetric Analysis (TGA4000, Perkin Elmer, Shelton, CT, USA) analysis was conducted to monitor the mass of the TPNF as a function of temperature or time during the calcination. TGA was used to quantify the decomposition or the loss of the PAN nanofiber template in the formation of THNF based on the weight of the nanofiber. Phase identification of the crystalline material was done using an X-ray diffractometer (XRD, Rigaku D/Max 2200 PC, Tokyo, Japan) with CuKα radiation (λ = 1.540 Å, 40 kV and 30 mA). Nitrogen adsorption/desorption measurement was performed to examine the surface area of the nanofiber samples with an automatic gas adsorption instrument (BEL, Belsorp-max, Osaka, Japan). A UV-Vis-NIR Spectrophotometer (UV-3101PC Shimadzu, Kyoto, Japan) was used in this study to measure the optical absorption behaviors of the photocatalyst. A similar instrument was used to measure the concentration of methylene blue (MB) in the solution at 664 nm wavelength.

### 2.5. Photodegradation Experiment

**Figure 1:**
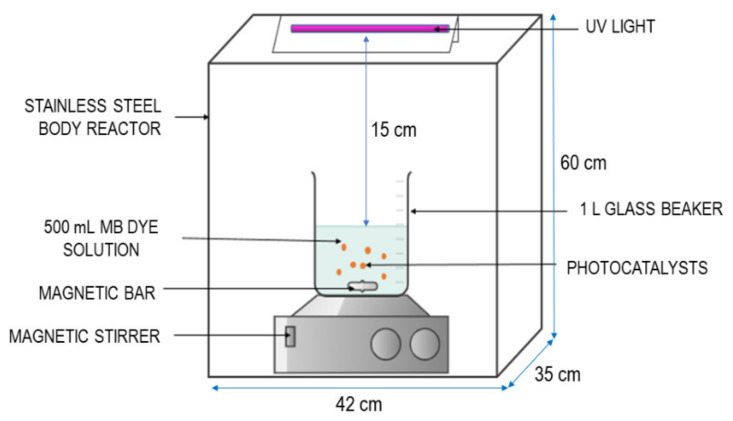
Schematic illustration of photocatalytic reactor.

Different concentrations of MB solution (i.e., 10, 20, 30, 40 and 50 mg/L) were prepared by dilution of the as-prepared 1000 mg/L MB stock solution. The photocatalytic degradation of MB dye using THNF photocatalysts was conducted under the illumination of a 3.0 mW/cm^2^ UV lamp (Vilber hourmat, λ = 312 nm, 30 watts) manufactured by Wuhan Co-shine Technology Co., Ltd., China, installed in a custom-made stainless steel photoreactor (60 × 35 × 42 cm^3^). The photoreactor consisted of a glass beaker, a magnetic stirrer, and the UV lamp was installed 15 cm above the beaker, as shown in Figure 1. In a typical photocatalytic degradation set-up, 0.25 g/L of THNF photocatalysts was suspended in 100 mg/L of model wastewater MB dye solution. The solution was dispersed using an ultrasonicator and oxygenated in the dark for 1 h to reach an adsorption–desorption equilibrium. At every 15 min interval, 5 mL of sample aliquot was collected to analyze the desorption percentage. After 1 h, the UV lamp was turned on while keeping the solution magnetically stirred. The sample was also collected at a regular time interval. The absorbance spectrum of the samples was measured using high-performance liquid chromatography (HPLC, Agilent Technology1200 Series). The degradation percentage was determined by Equation (1):

(1) $$Degradation\;\left(\%\right)=\frac{C_{0}-C}{C_{0}}\times 100=\frac{A_{0}-A}{A_{0}}\times 100$$

where *C*_0_ and *C* are the MB dye concentration initially and after irradiation, respectively. *A*_0_ and *A* are corresponding optical absorbances for initial and after irradiation, respectively.

## 3. Results

### 3.1. TiO_2_ Precursor Deposition on PAN Nanofiber

**Figure 2:**
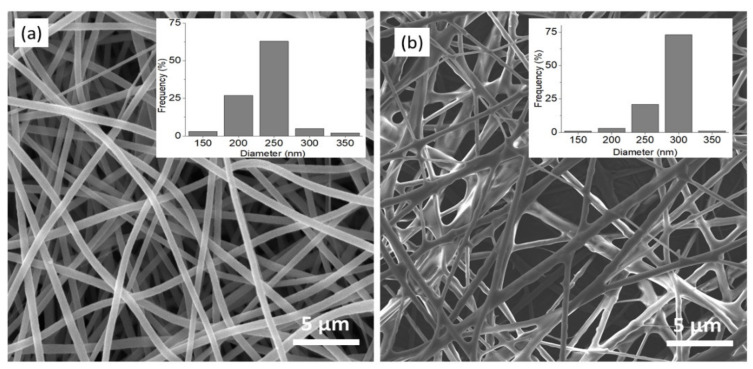
FESEM image of nanofibers and average diameter distribution (figure inset). (**a**) Electrospun PAN nanofibers; (**b**) TPNF nanofibers.

A successful synthesis of hollow nanostructures is influenced by the successful deposition of the target material precursor on the template. Morphology analysis on the PAN and TPNF was conducted using FESEM. Figure 2 shows the FESEM images and diameter distribution of the nanofibers. The electrospun PAN nanofibers have a randomly oriented, non-woven structure with a smooth surface. The average diameter of PAN nanofibers is 236 ± 16 nm. Meanwhile, the FESEM image of TPNF shows nanofibers with significantly rougher structures and larger diameters. The average diameter of TPNF is 286 ± 22 nm, as shown in the inset of Figure 2b. After the dip-coating of the TiO_2_ sol-gel precursor, the diameter of the nanofibers increased by about 20%. The dip-coating process had produced a uniform and conformal layer around the PAN nanofibers. The original well-defined structure and original arrangement of the PAN nanofibers are still retained after the TiO_2_ deposition.

**Figure 3:**
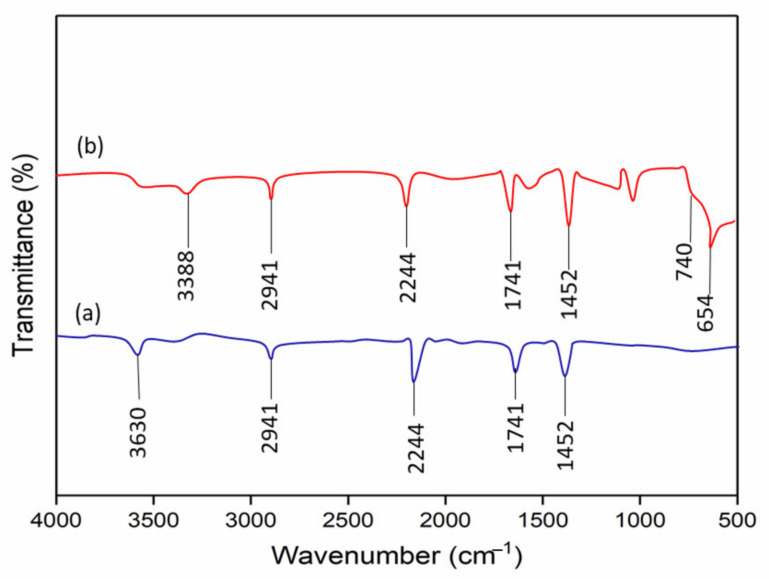
FTIR spectra of (**a**) Electrospun PAN nanofibers; and (**b**) TPNF nanofibers.

The deposition of TiO_2_ on PAN nanofibers was further confirmed by conducting FTIR analysis. The FTIR spectra of PAN nanofibers are shown in Figure 3a. The peaks that appeared at 3630 cm^−1^ can be attributed to –OH stretching vibrations. The peak at 2941 cm^−1^ was assigned to the stretching vibration of –CH in CH_2_. The peak at 2244 cm^−1^ was assigned to the stretching vibration of the C≡N functional group. The peak at 1741 cm^−1^ might originate from the stretching vibration of C=O, due to the hydrolyzed PAN or the residual DMF solvent. Meanwhile, the peak at 1452 cm^−1^ can be attributed to -CH bending vibration. This finding is in accordance with other published work by Zhang et al. [21]. The appearance of new peaks can be observed in the FTIR spectra of TPNF, as shown in Figure 3b. A broad peak appeared around 3000–3500 cm^−1^, which was believed to correspond to hydroxyl groups (–OH) in TiO_2._ The emerging broadband below 1000 cm^−1^ (740 and 654 cm^−1^) was ascribed to the characteristic of Ti–O and Ti–O–Ti stretching and bending vibration for TiO_2_. These observations confirm the successful deposition of TiO_2_ on the PAN template and imply that PAN is a compatible material to be used as the template in THNF synthesis.

**Figure 4:**
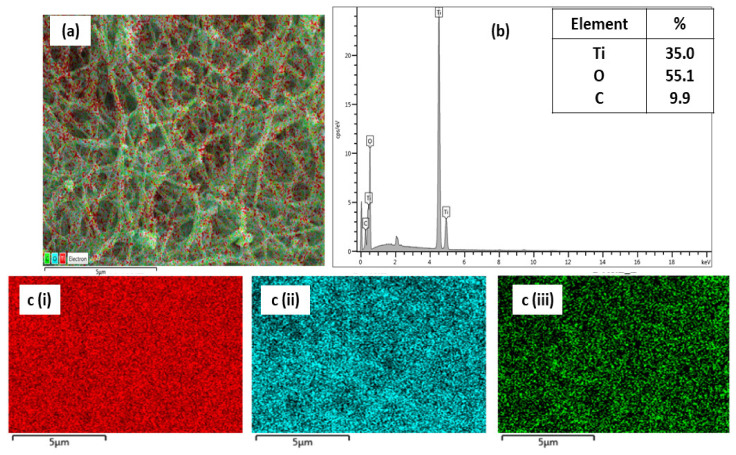
(**a**) Layered EDX mapping of TPNF; (**b**) EDX spectra of TPNF; (**c**) elemental mapping including (**i**)—Ti, (**ii**)—O, and (**iii**)—C.

Elemental analysis was also carried out to confirm the successful deposition of the TiO_2_ layer onto the PAN nanofibers. The composite nanofibers consisted of titanium (Ti), oxygen (O), and carbon (C) elements with an elemental percentage of 35.0%, 55.1%, and 9.9%, respectively. The EDX mapping in Figure 4a shows that Ti (red dots) was present uniformly on the nanofibers. This was confirmed by the EDX spectrum in Figure 4b, which shows that Ti and O were present in large quantities, while C was in a lower quantity. The relatively low percentage of C indicates that the majority of the surface of PAN nanofibers was covered with the TiO_2_ sol-gel. This result confirms the successful deposition of TiO_2_ on PAN nanofibers.

### 3.2. Effect of Calcination Temperature

#### 3.2.1. Thermal Decomposition Analysis

**Figure 5:**
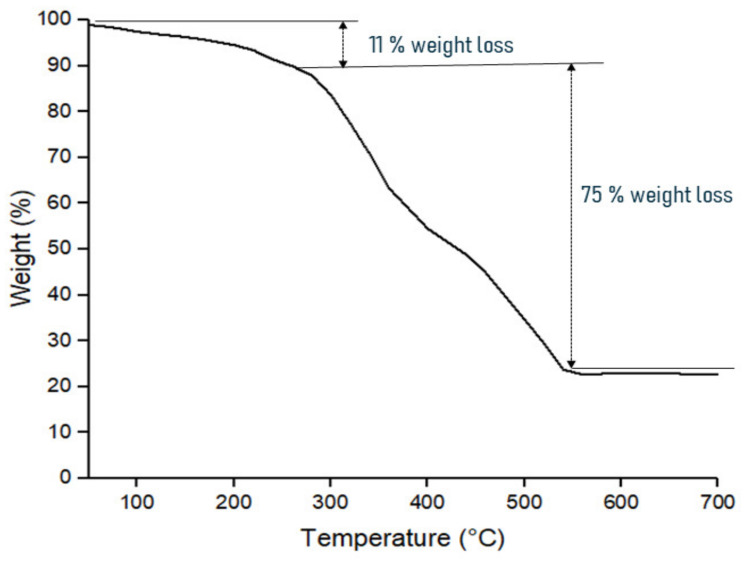
TGA curve of TPNF nanofiber decomposition.

TPNF was calcined at a high temperature between 400 to 600 °C to obtain THNF. The effect of temperature on the decomposition of TPNF was analyzed by using thermogravimetric analysis (TGA). The curve of weight loss percentage against temperature is presented in Figure 5. It can be observed that the decomposition of TPNF occurred in three stages. From the starting of the heating process up to 280 °C, the weight of the nanofibers slowly decreases by about 11%. This behavior is associated with the loss of moisture in hydrous TiO_2_ and the evaporation of DMF solvent. From 280 to 540 °C, the nanofibers undergo rapid weight loss by about 77% from the original weight of TPNF. The weight loss is due to the breaking of the C-C bond and decomposition of the PAN component. A previous study confirmed that the melting point of polyacrylonitrile is 317 °C, and it decomposes beyond that [22]. Through mass spectrometry analysis, absolute confirmation of the decomposition is made by identifying the gaseous products as they evolved. Hydrogen cyanide (HCN) was identified as the predominant product, while ammonia (NH_3_) existed in a smaller quantity. Finally, a stagnant weight of TPNF is observed beyond 540 °C, indicating that the PAN template has been removed completely. These findings are similar to the TGA analysis by Gao et al. [23] for the decomposition of PVA nanofibers. Therefore, the total weight loss of TPNF was about 86% from its original weight. These findings indicated that THNF is successfully formed at a temperature of 600 °C.

#### 3.2.2. Morphological Analysis

**Figure 6:**
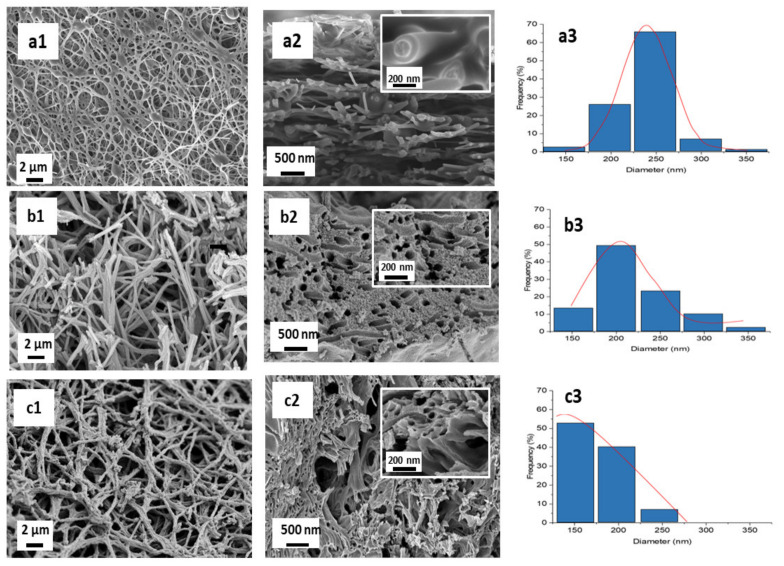
FESEM morphologies of THNF calcined at different temperatures; (**1**) 5000 magnification; (**2**) 10,000 magnification; (**3**) diameter distribution where (**a**) THNF400, (**b**) THNF500, and (**c**) THNF600.

Figure 6(a1) shows the FESEM images of THNF400 at 5k magnification. The nanofibers have a wriggly and irregular surface. The nanofibers calcined at 400 °C have good interconnectivity with each other. Figure 6(a2) shows the cross-sectional FESEM image of the nanofibers at 10k magnification. In the figure inset of Figure 6(a2), the boundary between the inner and outer sections is visible. However, no hollow structured nanofibers can be observed at this temperature. The average nanofiber diameter is 236 ± 34 nm, as shown in Figure 6(a3). These observations imply that at 400 °C, PAN nanofibers had melted and decomposed, corresponding to the TGA curve in Figure 6. Meanwhile, TiO_2_ nanoparticles around the PAN nanofibers had also been formed. However, the appearance of TiO_2_ was not obvious, possibly because of the melted PAN nanofibers that covered the surface of the nanofibers. The agglomeration of melted PAN nanofibers and the formed TiO_2_ nanoparticles is a possible explanation for the wriggly and irregular surface of the nanofibers. The synthesis of titanium dioxide hollow nanofibers was not successful at 400 °C because it was not sufficient to completely burn off PAN nanofibers. Hence, the calcination temperature was increased to 500 °C.

Figure 6(b1) reveals THNF500 with a rougher surface and shorter length relative to THNF400, with an average diameter of 240 ± 39 nm. Referring to the TGA curve in Figure 5, at 500 °C, the weight of the nanofibers remains unchanged, which implies that most of the PAN components had been removed. The disappearance of PAN reveals the TiO_2_ nanoparticles surrounding the nanofibers. The rougher surface is attributed to the promoted growth of crystalline TiO_2_ grain as the temperature increased. The heat effect also causes a decrease in nanofiber length. Figure 6(b2) shows the appearance of hollow cavities at the nanofibers cross-section, with agglomerated nanofibers strands.

The formation of THNF was studied at the temperature of 600 °C. Figure 6(c1) shows the FESEM image of THNF600 at 5k magnification. In comparison to THNF500, the surface of THNF600 was prominently rougher, due to the highly crystalline properties caused by the increasing calcination temperature. The average diameter of THNF600 was 177 ± 32 nm. The decrease in diameter can be attributed to the shrinkage of nanofibers during the heating process. In Figure 6(c2), hollow cavities at the nanofibers core can be observed. Therefore, it can be deduced that the formation of TiO_2_ hollow nanofibers is highly dependent on the decomposition of PAN and the formation of TiO_2_ nanoparticles on the shell side of the PAN fibers, which are influenced by the increase in the calcination temperature.

#### 3.2.3. Nitrogen-Desorption Analysis

Degussa P25 is a commercially available titanium dioxide photocatalyst that is widely used, attributed to its high level of photocatalytic activity. However, one of the remaining issues of this type of photocatalyst is its low specific surface area (S_BET_), which is about 50 m^2^/g. Hence, with the synthesis of hollow-structured TiO_2_ nanofibers, we aimed to further enhance the specific surface area of the catalyst. The specific surface area and porosity of THNF400, THNF500, and THNF600 were quantified using nitrogen adsorption–desorption by applying the Brunauer, Emmett, and Teller (BET) theory.

The BET surface area of each sample is tabulated in Table 1. THNF400 is observed to have an S_BET_ of only 13.3216 m^2^/g. This value is significantly lower than that of Degussa P25. Meanwhile, the specific pore volume of THNF400 was 0.06354 cm^3^/g. In reference to Figure 6, at 400 °C, the majority of the PAN component still existed in the nanofibers. Therefore, the low surface area and pore volume of THNF400 could possibly be caused by the melting of the PAN component that covered and clogged the existing pores on the surface of the nanofibers. This statement is corroborated by the FESEM image of THNF400 in Figure 6. On the other hand, relatively higher S_BET_ and specific pore volume were recorded by THNF500, which was 43.4085 m^2^/g and 0.2349 cm^3^/g, respectively. The increased value is possibly due to the almost completed PAN decomposition at 500 °C, which led to the appearance of hollow cavities and the revelation of the rougher structure of the nanofibers. With the increasing calcination temperature, the effect of heat produces more crystalline TiO_2_ with more prominent grains. In the presence of hollow cavities and rough surfaces, there was a higher available surface for nitrogen adsorption. THNF600 possessed the highest S_BET_ and specific pore volume, which were 81.2776 m^2^/g and 0.32716 cm^3^/g, respectively. Based on the morphology and average diameter of THNF600, the increase of S_BET_ and specific pore volume can be attributed to the hollow structure and smaller diameter of the nanofibers. Hollow-structured nanofibers consist of outer walls and empty spaces inside a distinct shell. The abundance of reactive sites increases the area for nitrogen adsorption. This phenomenon is in line with a previous study that stated that the surface area for hollow copper oxide particles is larger than the solid particles of corresponding material [24]. Meanwhile, the relatively smaller diameter of THNF600 gives it a higher surface area-to-volume ratio. The smaller the diameter of the nanofibers, the larger the adsorption area per unit volume. This property gives THNF600 the upper hand in photocatalytic reactions.

#### 3.2.4. Crystallinity Analysis

**Figure 7:**
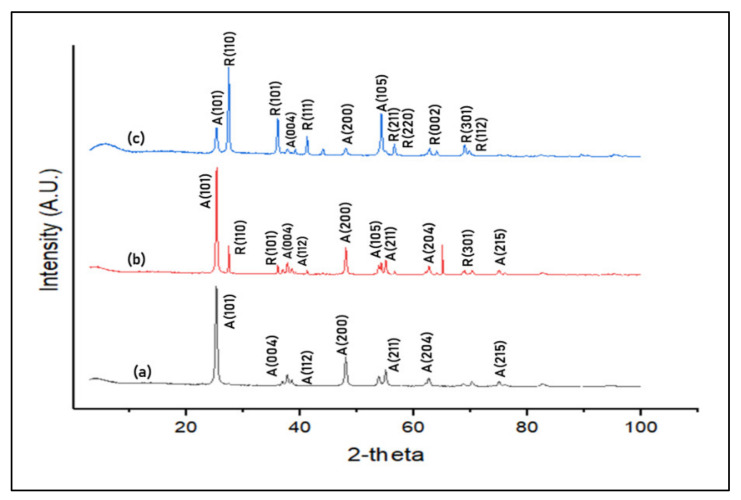
XRD patterns. (**a**) THNF400, (**b**) THNF500, (**c**) THNF600.

The identification of the phases that exist in THNF400, THNF500, and THNF600 was conducted using X-ray diffraction (XRD). Based on the XRD patterns presented in Figure 7, it can be deduced that all samples are crystalline. From XRD spectra (a), the peaks of THNF400 are identified at 2Ɵ = 25.3°, 37.7°, 41.2°, 48.0°, 55.0°, 62.7°, and 75.0°, which correspond to (101), (004), (112), (200), (211), (204), and (215) crystal planes. Based on JCPDS card no. 21-1272, all the diffraction peaks are well-defined and can be assigned to the anatase TiO_2_. Since there are no other peaks associated with other crystalline forms were detected, this indicates that THNF400 was purely anatase.

The transformation of the anatase to rutile phase can be seen in Figure 7, XRD spectra (b). A mixture of anatase and rutile diffraction peaks can be observed in THNF500. Rutile TiO_2_ begins to form with the emerging of relatively weak rutile diffraction peaks at 2Ɵ = 27.4°, 36.0°, and 68.7°. These peaks correspond to (110), (101), (301) planes based on JCPDS card no. 21-1276. Meanwhile, the other peaks are (101), (004), (112), (200), (105), (211), (204) and (215), which existed at 2Ɵ = 25.3°, 37.7°, 41.2°, 48.0°, 53.7°, 55.0°, 62.7° and 75.0°. Upon calculation, THNF500 consisted of 82.1% anatase and 17.9% rutile TiO_2_.

Meanwhile, further crystallization is observed in THNF600, XRD spectra (c), which is comprised of 24.2% anatase and 75.8% rutile. Additional rutile peaks begin to emerge at 2Ɵ = 41.2°, 56.6°, 56.8°, 62.8° and 69.7°, which corresponds to the planes (111), (211), (220), (002), and (112). The existing rutile peaks of (110), (101), (301) planes had increased in sharpness and intensity.

From the XRD result, it can be concluded that the phase conversion from anatase to rutile is highly influenced by the calcination temperature. Upon heat treatment, the octahedral TiO_2_ undergoes distortion, in which the breaking and reforming of new Ti-O bonds takes place at the grain interface and interior. The bond breaking and lattice distortion resulted in oxygen vacancies. The oxygen vacancies serve as a nucleation site that facilitated the formation of the rutile phase [25].

The Scherrer equation was employed to estimate the crystallite size of the photocatalysts [26]. The crystallite size of THNF increased from 15.67, 21.45 to 30.03 nm as the calcination temperature increased. Higher calcination temperature had reduced the activation energy, thus promoting the crystal growth rate [27]. It was commonly known that the surface area is inversely proportional to the crystallite size because there is a greater proportion of crystals and more area to occupy [28]. However, in this study, THNF400, which had the smallest crystallite size, possessed a BET surface area of only 0.06354 m^2^/g, which was relatively low compared to those of THNF500 and THNF600. This phenomenon could be explained by the physical structure of the nanofibers itself, as observed in Figure 6. Despite having a larger crystallite size, THNF600 had a larger BET surface area due to the comparatively rougher structure, the presence of hollow cavities, and less agglomerated nanofiber strands. These enhanced surface properties help for better catalytic effects to speed up the surface reactions between different adsorbed reactants species and TiO_2_ catalysts.

#### 3.2.5. Optical Absorbance Analysis

**Figure 8:**
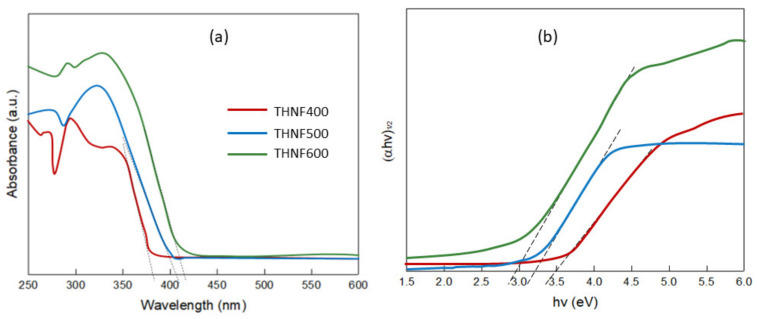
(**a**) UV–Vis diffuse reflectance spectra and (**b**) Tauc plot for bandgap determination.

The optical properties of the photocatalysts were evaluated using a UV-Vis spectrophotometer. The UV-Vis spectrum of THNF400, THNF500, and THNF600 is shown in Figure 8a. The absorption edges of THNF400, THNF500, and THNF600 were located at about 381, 403, and 422 nm, respectively. THNF400 showed an absorbance stopping edge in the UV region (<400 nm). The absorption band at 381 nm can be assigned to the bandgap excitation of anatase TiO_2_ that corresponds to the band-to-band transition from the Ti *3d* level to O *2p* levels [29]. At a higher calcination temperature, there was a noticeable “redshift”, as indicated by the presence of shoulder in the spectra of THNF500 and THNF600 near the visible region. The enhancement in the absorption is possibly due to the presence of rutile content that intrinsically has a smaller bandgap in comparison to the pristine anatase phase [30].

The bandgap energy of the photocatalysts was estimated using the Kubelka–Munk function. A graph of (αhν)^1/2^ against the energy of absorbed light (eV) was plotted, as shown in Figure 8b. The bandgap energy was found to decrease from 3.50, 3.24, and 3.00 eV as the calcination temperature increased. These results indicate that THNF600 has better optical properties than THNF400 and THNF500.

It is generally known that rutile TiO_2_ has a narrower bandgap in comparison to anatase due to its structural properties. TiO_2_ consists of Ti^4+^ surrounded by six oxygen ions forming TiO_6_^2−^. The effect of heat during calcining had altered the arrangement of ions in the TiO_2_ nanoparticles, thus increasing the fraction of rutile in the catalysts. As the Ti-Ti distance in rutile is shorter than in anatase, the rutile TiO_2_ is 9% denser. Therefore, this has caused more pronounced orbital localization of the Ti 3*d* and O 2*p* and resulted in a narrower bandgap [31]. In accordance with the XRD patterns in Figure 7, the bandgap narrowing was attributed to the higher crystallinity of THNF600, in which the majority of the fraction was rutile (75.8%). The mixed-phase photocatalysts had created heterojunction between the interface of the anatase and rutile phase. The heterojunction between the two phases accelerates the electron and hole separation due to the difference in the bandgap energy of the two phases. This phenomenon causes a difference in electric potential, which creates a strong electric field to accelerate electron and hole separation.

#### 3.2.6. Photocatalytic Degradation of MB Dye

**Figure 9:**
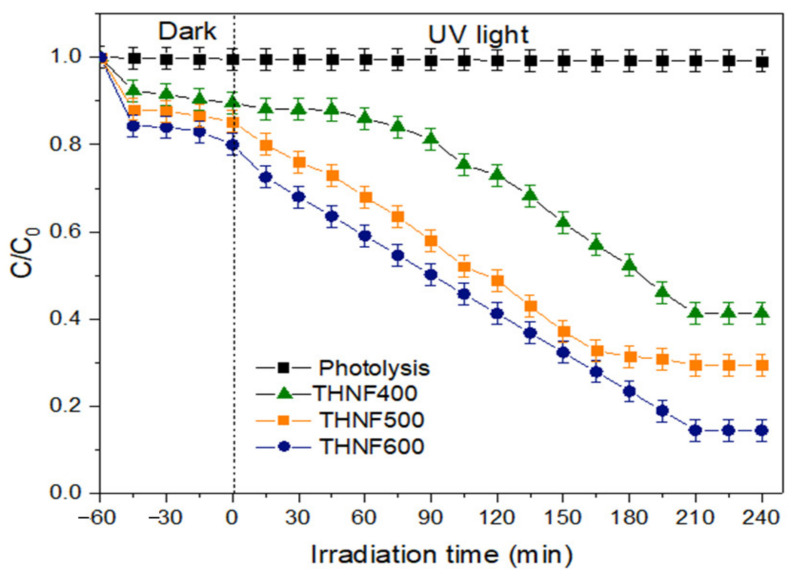
Degradation of MB dye with different catalysts (Catalyst loading = 0.50 g/L, MB dye concentration = 10 ppm, pH of solution = 7).

Figure 9 demonstrates MB dye degradation in an aqueous solution from the photocatalytic degradation test using THNF400, THNF500, and THNF600 photocatalysts as a function of irradiation time. The photocatalytic activity of the prepared catalysts was measured by the concentration of MB dye. The photolysis experiment using no catalysts was also set up as a control.

The degradation process was run for 5 h, including 1 h of the adsorption process in the dark. In the photolysis process, the degradation of MB dye was not significant (<5%). The degradation percentage of MB dye using THNF400, THNF500, and THNF600 was 42.9%, 61.7%, and 85.5%, respectively. Therefore, it can be deduced that the degradation of MB dye is subjected to the presence of a photocatalyst.

**Figure 10:**
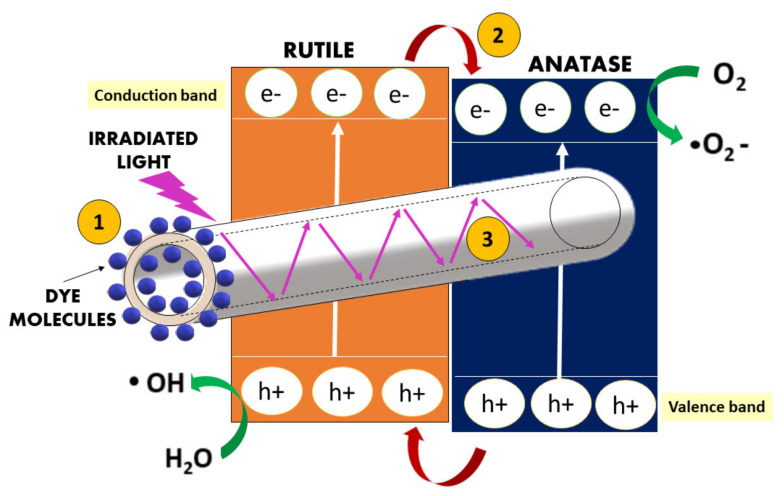
Titanium dioxide hollow nanofibers with enhanced photocatalytic properties. (1) The additional surface provided by the inner core and outer shell for molecule adsorption, (2) heterojunction created by mixed-phase photocatalyst, and (3) light scattering effect within the nanofiber interior.

The photocatalytic performance of THNF600 has significantly outperformed THNF400 and THNF500. With regard to the analysis of photocatalysts properties, the excellent performance of THNF600 can be ascribed to: (i) the extended surface area for UV irradiation and pollutant molecule adsorption; (ii) accelerated electron-hole separation due to heterojunctions between the anatase and rutile phase; and (iii) efficient light utilization due to light scattering effect in hollow nanofibers, as illustrated in Figure 10.

THNF600 is made up of rough-surfaced nanofibers with hollow cavities. These had caused THNF600 to possess a larger surface area, in accordance with the S_BET_ result. Generally, the degradation of pollutants occurs on the surface of the photocatalysts, so photocatalysts with larger adsorption sites are more favorable for the particular reaction. The large accessible surface area of THNF600 allows a higher amount of MB dye molecules to be diffused towards the THNF surface and consequently adsorbed at the active sites within the inner and outer spaces of the hollow nanofibers. The larger surface area of THNF600 also provided more active sites for UV illumination, resulting in higher production of •OH radicals. Besides, the hollow nanofiber structure of THNF600 had also improved the degradation process in terms of charge separation efficiency. The presence of shells and hollow cavities within THNF600 had reduced the migration distance of the photoexcited electron, which suppressed the electron-hole recombination.

Apart from that, hollow-structured photocatalysts have better light scattering effect as compared to dense photocatalyst nanofiber. Photocatalyst nanofiber with a regular dense structure tends to strongly reflect light, so light can only be adsorbed within the scale of the particle size. This leads to poor light utilization. In a hollow structure, as light penetrates the shell of the catalysts, the incident light is scattered strongly within the interior of nanofiber. The secondary light adsorption enhanced the light-harvesting properties of the catalyst. The light scattering effect had contributed to the efficient utilization of photogenerated charge carriers, thus increasing the photodegradation of MB dye using THNF600.

As can be seen from the UV-Vis absorption spectrum (Figure 8), the bandgap of THNF600 that is lower than those of THNF400 and THNF500 also contributes to the higher photocatalytic performance. The narrow bandgap had eased the transfer of electrons from VB to CB. This remarkable bandgap narrowing is attributed to the synergistic effect of the anatase and rutile phase presented in THNF600. In mixed-phase photocatalysts, heterojunction between the two crystalline lattices was created, resulting in efficient electron-hole separation. Therefore, based on the performance of different catalysts, THNF600 was used for the next experiments.

(a) Effect of photocatalyst loading

**Figure 11:**
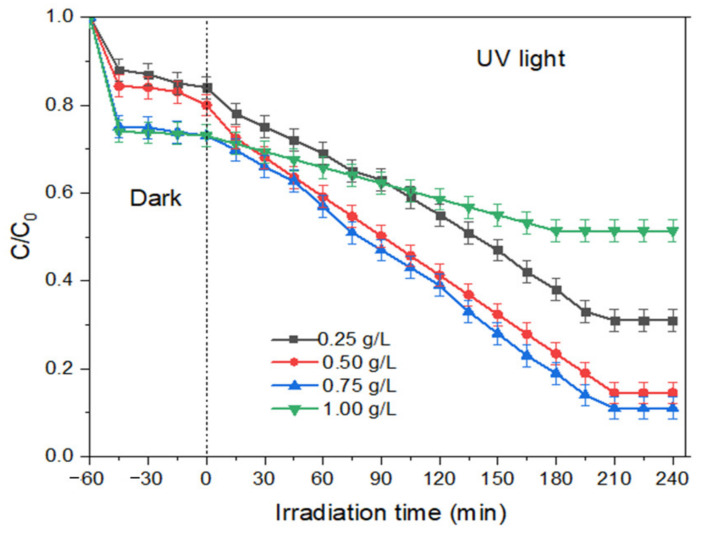
Effect of photocatalyst dosage on degradation efficiency of MB dye.

The determination of optimum photocatalyst dosage is essential because it affects the economics of the process, especially in the scaling-up process of the reaction. Other than that, the amount of catalyst used is also significant in the downstream processing, where the catalyst needs to be separated from the reaction medium. For the degradation of MB dye using THNF, the dosage of photocatalysts was studied in between the range of 0.25 to 1.0 g/L. Meanwhile, the MB dye concentration and pH were kept constant at 10 ppm and pH 7. As can be observed in Figure 11, the efficiency of MB dye degradation increases with increasing photocatalyst loading. The highest degradation percentage was obtained with 0.75 g/L THNF600, which is 85.5%. In the presence of more photocatalyst, the active sites of catalyst are higher, which increases the amounts of photons adsorbed as well as the chances for hydroxyl radical production, allowing a higher number of MB dye molecules to be mineralized. However, the degradation efficiency decreases to 49% at 1.0 g/L catalyst loading. This phenomenon is possibly caused by the interception of light in the presence of a high amount of suspended catalyst. This is also in agreement with the previous work for the degradation of phenol using N,S co-doped TiO_2_ under visible light irradiation [32].

(b) Effect of dye concentration

**Figure 12:**
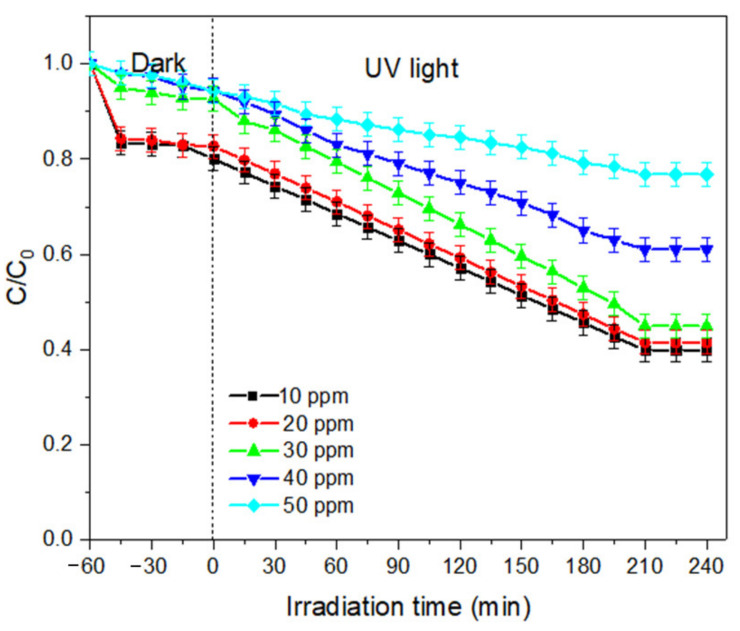
Effect of initial dye concentration on degradation efficiency of MB dye.

The determination of optimum initial BPA concentration is important to overcome the mass transfer deterrent force between the liquid and solid phases. In this study, the impact of increasing initial MB dye concentration was studied within the range of 10 to 50 ppm. The effect of initial BPA concentration on degradation efficiency is presented in Figure 12. A significant reduction of degradation efficiency can be observed as the MB dye concentration increases. When the concentration of pollutants increases, more of the pollutant molecules are adsorbed on the surface of the photocatalysts. Hence, this had caused the accumulation of pollutants and competition for active sites. The irradiated UV is mostly adsorbed by the pollutant molecules instead of the catalysts, resulting in lower production of ·OH radicals. Therefore, with an increasing concentration of MB dye, the number of free radicals attacking the MB dye molecules decreases. We can agree that with increasing concentration of MB dye, a higher amount of catalyst loading is required [33].

(c) Effect of pH of the solution

**Figure 13:**
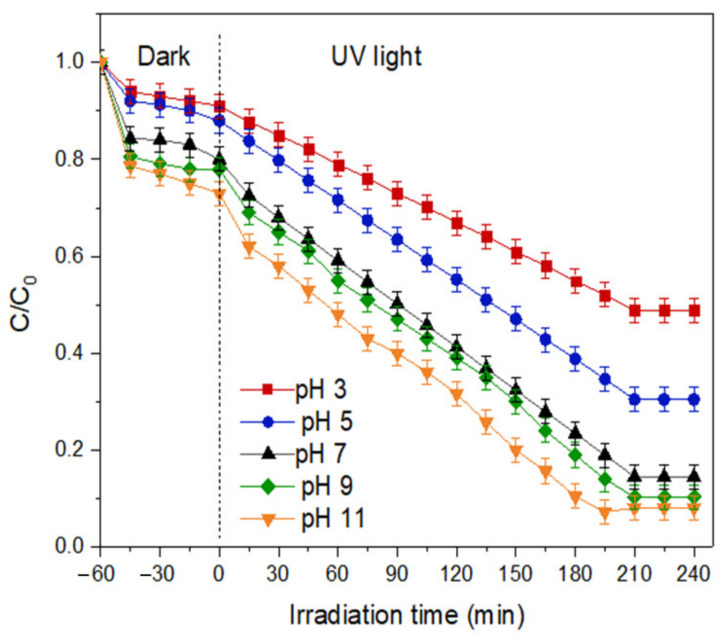
Effect of pH on degradation efficiency of MB dye.

The degradation of MB dye using THNF600 catalysts was examined in the pH range of 3 to 11. NaOH and HCl were used to adjust the pH accordingly. The results in Figure 13 show that the highest degradation of MB dye occurs at pH 11. The change in pH of the solution affected the surface charge of a photocatalyst material due to the ionization of titania, as shown in Equations (2) and (3).

TiOH + H^+^ → TiOH^2+^ (2)

TiOH + OH^−^ → TiO^−^ + H_2_O (3)

In acidic conditions, TiO_2_ is protonated upon receiving H+ from the medium, therefore it is positively charged. Meanwhile, in the basic medium, TiO_2_ is deprotonated, making it negatively charged [34]. Due to the Coulombic force between the cationic MB dye molecules and the positively charged THNF600 surface, repulsion between the molecules and photocatalysts occurs at low pH, hence a lower degradation percentage is expected. On the other hand, at higher pH, the cationic MB dye molecules are attracted to the negatively charged THNF600 surface, causing a better degradation percentage. The corresponding results can be seen in the previous study for the degradation of MB dye [35].

(d) Effect of hydrogen peroxide concentration

**Figure 14:**
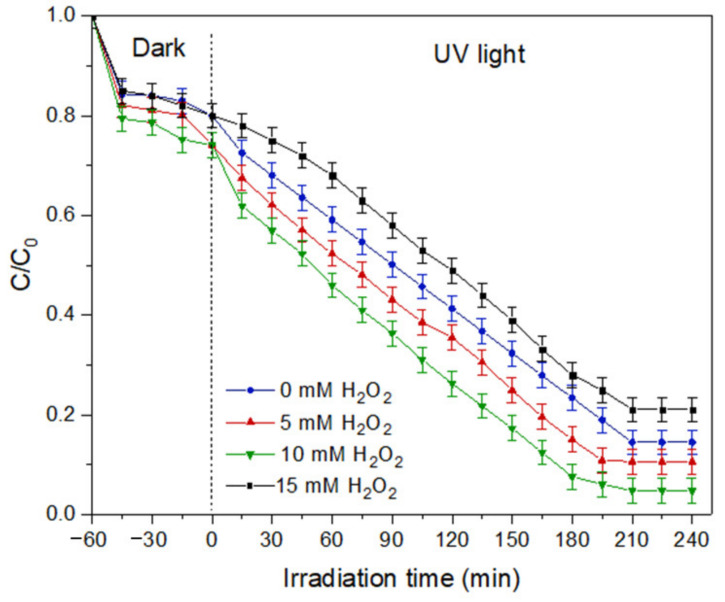
Effect of hydrogen peroxide concentration on degradation efficiency of MB dye.

The effect of hydrogen peroxide concentration in the solution was investigated by using 5, 10, and 15 mM of H_2_O_2._ At low H_2_O_2_ concentration, the degradation of MB achieved 89.35%, as portrayed in Figure 14. The addition of H_2_O_2_ to 10 mM can improve the degradation percentage to 95.2% by increasing the production of hydroxyl radicals. Upon the irradiation of light energy, the O-O bonds in the H_2_O_2_ are broken and create active radicals that are used to mineralize the MB dye molecules. However, the result shows decreasing MB dye degradation with 15 mM H_2_O_2_. A high concentration of H_2_O_2_ performs as an electron scavenger. The excessive amount of H_2_O_2_ tends to react with the hydroxyl radicals, causing competition with the pollutant molecules to be mineralized, as has been reported previously [36].

### 3.3. Proposed Mechanism of Hollow Nanofibers Formation

The fabrication of hollow nanofibers using the template synthesis technique is based on the successful deposition of target material on the template and also the elimination of the template. In this study, the PAN nanofiber template was coated with TiO_2_ sol-gel. Essentially, sol-gel is commonly used to synthesize nanoparticles in the presence of the precursor alkoxides. An acid or a base is usually added to assist the hydrolyzation of the precursor. Nanoparticle formation from the alkoxides involve hydrolysis, condensation, further condensation, and is followed by the growth of nanoparticles. Meanwhile, the elimination of the template is done through physical and chemical approaches, including calcination, dissolution, and etching. It is crucial to study how the calcination temperature affects the formation of THNF. Apart from removing the PAN via decomposition under high temperatures, the calcination process also affected the physicochemical properties of the THNF, as discussed in the previous section.

**Figure 15:**
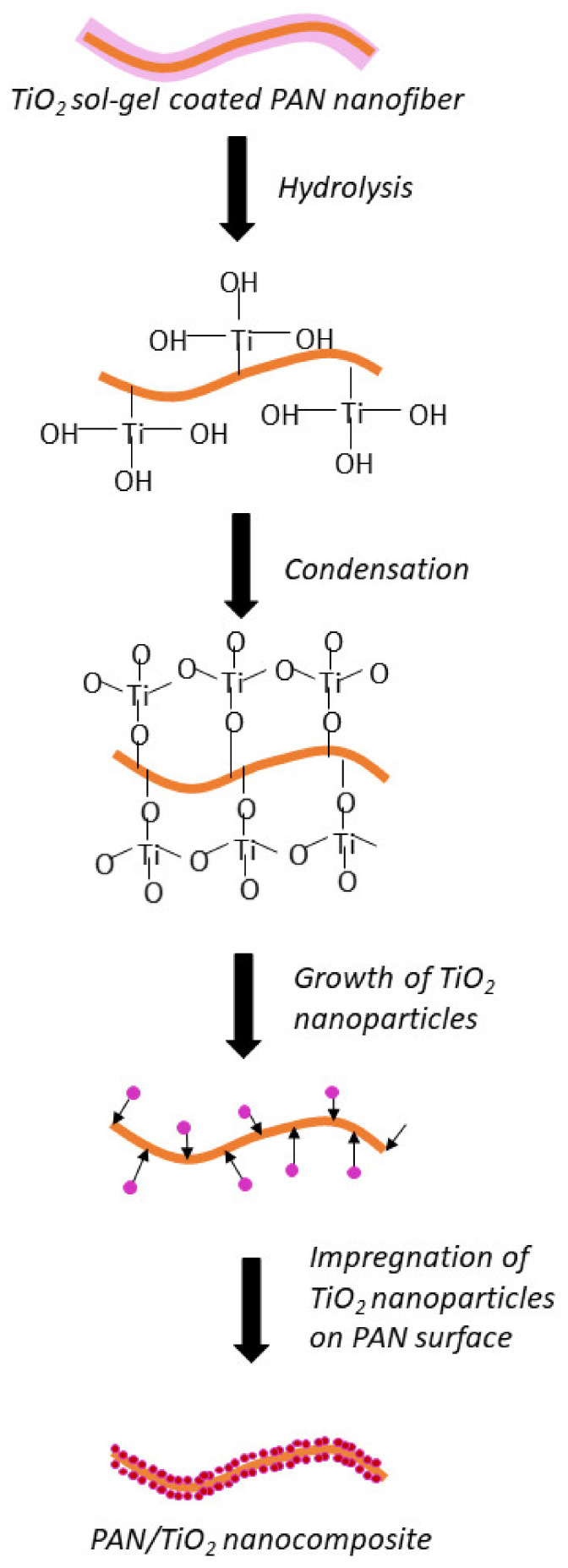
The mechanism for the nucleation and growth of the TiO_2_ nanoparticles on the surface of PAN nanofibers.

Generally, TiO_2_ is prepared by hydrolysis and condensation of titanium alkoxides, Ti(OR)_n_. Particularly in this study, titanium isopropoxide Ti[OCH(CH₃)₂]_4_ was used as the precursor. A hydrolysis reaction occurs when the Ti-OR group is substituted with water to Ti-OH moieties, as presented in Equation (4). Then, the condensation process forms Ti-O-Ti or Ti-OH-Ti oxide networks, as shown in Equation (5). Nucleation and growth of TiO_2_ particles occur when the oxide network undergoes further condensation, as shown in Equation (6). Since the TiO_2_ sol-gel has been successfully coated on the PAN nanofibers, the obtained TiO_2_ nanoparticles impregnate the surface of PAN nanofibers, forming a TPNF nanocomposite. The overall reaction is shown in Equation (7). The mechanism of TiO_2_ nanoparticle growth on the PAN surface is illustrated in Figure 15.

Hydrolysis of TTIP:

Ti[OCH(CH_3_)_2_]_4_ + 4H_2_O → Ti(OH)_4_ + 4[CH(CH_3_)_2_OH] (4)

Condensation of Ti(OH)_4_:

2[Ti(OH)_4_] → Ti(OH)_3_-O-Ti(OH)_3_ (5)

Further condensation:

Ti(OH)_3_-O-Ti(OH)_3_ → 2TiO_2_ + 3H_2_O (6)

Overall reaction:

Ti[OCH(CH_3_)_2_]_4_ + 2H_2_O → TiO_2_ + 4[CH(CH_3_)_2_OH] (7)

## 4. Conclusions

In this work, TiO_2_ hollow nanofibers have been successfully synthesized via template synthesis. The obtained hollow nanofibers exhibit admirable photocatalytic performance attributed to the anatase–rutile heterojunction creation, extended surface area for UV and dye molecules adsorption, and light-harvesting effect in the hollow cavities of the nanofibers. The calcination temperature plays an important role in the formation of the hollow structures and in determining the physicochemical properties of the TiO_2_ hollow nanofibers. The TiO_2_ hollow nanofibers were successfully synthesized at 600 °C and THNF600 has the best characteristics in terms of morphology, surface area, crystallinity, and optical properties to be used for the photodegradation process. We had also investigated the effects of operating parameters on the photodegradation of MB dye solution. The highest photocatalytic degradation efficiency was obtained at catalyst loading = 0.75 g/L, MB dye concentration = 10 ppm, pH = 11 and H_2_O_2_ concentration = 10 mM. Lastly, the mechanism of TiO_2_ hollow nanofiber formation was also proposed.

## Figures and Tables

**Table 1:** Specific surface area and pore volume of the nanofibers.

Sample	Specific Surface Area (m^2^/g)	Specific Pore Volume (cm^3^/g)
THNF400	13.3216	0.06354
THNF500	43.4085	0.23490
THNF600	81.2776	0.32716

## Data Availability

Not applicable.
